# Diabetes-Related Quality of Life Assessment in Children following Total Pancreatectomy with Islet Autotransplantation

**DOI:** 10.1155/2023/2851620

**Published:** 2023-06-22

**Authors:** Jacob M. Redel, Lindsey Hornung, Deborah Elder, Jaimie D. Nathan, Sarah Corathers, Kristin L. Rich, Maisam Abu-El-Haija

**Affiliations:** ^1^Department of Pediatrics, University of Cincinnati College of Medicine, Cincinnati, Ohio, USA; ^2^Division of Endocrinology, Cincinnati Children's Hospital Medical Center, 3333 Burnet Ave, Cincinnati, Ohio 45229, USA; ^3^Department of Pediatrics, University of Missouri-Kansas City School of Medicine, Kansas, Missouri, USA; ^4^Division of Endocrinology, Children's Mercy Hospital, Kansas, Missouri, USA; ^5^Division of Biostatistics and Epidemiology, Cincinnati Children's Hospital Medical Center, 3333 Burnet Ave, Cincinnati, Ohio 45229, USA; ^6^Department of Surgery, University of Cincinnati College of Medicine, Cincinnati, Ohio, USA; ^7^Division of Pediatric General and Thoracic Surgery, Cincinnati Children's Hospital Medical Center, 3333 Burnet Ave, Cincinnati, Ohio 45229, USA; ^8^Department of Abdominal Transplant and Hepatopancreatobiliary Surgery, Nationwide Children's Hospital 700 Children's Drive, Columbus, Ohio 43205, USA; ^9^Department of Surgery, The Ohio State University Wexner Medical Center 395 West 12th Avenue, Columbus, Ohio 43210, USA; ^10^Division of Behavioral Medicine and Clinical Psychology, Cincinnati Children's Hospital Medical Center, Cincinnati, Ohio 45229, USA; ^11^Division of Gastroenterology, Hepatology and Nutrition, Cincinnati Children's Hospital, OH USA Medical Center, 3333 Burnet Ave, Cincinnati, Ohio 45229, USA

## Abstract

Total pancreatectomy with islet autotransplantation (TPIAT) can improve pain and reduce functional impairment associated with acute recurrent or chronic pancreatitis. However, long-term glucose monitoring and insulin therapy are often required, which can adversely affect the quality of life. We sought to evaluate diabetes-related quality of life (DR-QOL) in youth who underwent TPIAT and compare it to the youth with new-onset type 1 diabetes (T1D). The Pediatric Quality of Life Inventory™ 3.2 Diabetes Module (PedsQL™ DM) was used to assess DR-QOL in 46 youth (<20 years old) who underwent TPIAT. The PedsQL™ DM scores were analyzed for statistically significant changes and minimally important clinical differences (MCID) over time post-TPIAT. Scores at 12 months (*n* = 29) and 24 months (*n* = 16) were then compared to PedsQL™ DM scores from a historical cohort of demographically similar (age and sex) youth with a 12 months (*n* = 52) and 24 months (*n* = 58) after diagnosis of T1D. The diabetes symptoms summary score (mean 65 to 57 and *p*=0.03) and the total score (mean 74 to 68 and *p* < 0.05) decreased (worsened) during the first 24 months post-TPIAT and met the MCID threshold, suggesting the decrease in these scores was clinically significant. Post-TPIAT PedsQL™ DM scores were not significantly different than youth new diagnosis of T1D after 24 months (all *p* > 0.2). In youth who underwent TPIAT, DR-QOL worsened over the first two years, mostly attributable to the diabetes symptoms score. Compared to children with T1D, post-TPIAT DR-QOL was similar two years after diabetes onset.

## 1. Introduction

Total pancreatectomy with islet autotransplantation (TPIAT) can alleviate pain and functional impairment associated with acute recurrent pancreatitis (ARP) or chronic pancreatitis (CP) in children [1–3]. This operation is an option for patients who have severely impaired quality of life from ARP or CP and in whom other interventions have not been successful. TPIAT involves surgical resection of the pancreas, isolation of islet cells, and injection of the autologous islets into the portal vein for engraftment into the liver. Improvements in physical and mental health, as well as overall quality of life, have all been demonstrated in children after this procedure [3–5]. However, diabetes-specific patient-reported outcome measures, such as diabetes-related quality of life (DR-QOL), in youth undergoing TPIAT have not yet been assessed.

DR-QOL scores have been shown to be lower in children with type 1 diabetes (T1D) compared to healthy peers[6], but data on DR-QOL in youth after TPIAT are lacking. A subset of children undergoing TPIAT will develop postpancreatectomy diabetes and require chronic insulin therapy [1]. However, post-TPIAT glycemic control and insulin requirements can be variable, and it can be difficult for clinicians and families to conceptualize exchanging the symptoms of ARP/CP for the burden of diabetes management. Given the possibility of decreasing insulin requirements and improving glycemic control over time post-TPIAT, we hypothesized that DR-QOL may gradually improve and that post-TPIAT DR-QOL would eventually be better than is seen in children with T1D. Therefore, our aims were to: (1) objectively evaluate DR-QOL over time in youth who underwent TPIAT and (2) compare DR-QOL in youth who underwent TPIAT with demographically similar peers recently diagnosed with T1D.

## 2. Materials and Methods

### 2.1. Study Design

For this retrospective cohort study, we utilized PedsQL™ DM survey data collected periodically at post-TPIAT follow-up clinic visits to evaluate for a change in scores over time using linear regression models. Cross-sectional survey data at 12  and 24 months were then compared to survey data from a cohort of T1D patients at the same institution 12 and 24 months after diabetes onset. The T1D survey results were limited to the 18 diabetes management questions that were routinely collected for clinical care. The diabetes management summary score and subscale scores were compared between the groups. This study was approved by the Cincinnati Children's Hospital Institutional Review Board.

### 2.2. Recruitment of TPIAT Participants

Patients undergoing TPIAT at the Pancreas Care Center of Cincinnati Children's Hospital Medical Center were enrolled in this study prior to surgery. All participants aged 18 years or older provided informed written consent. Participants who were 11–17 years old provided written assent and parents of all participants <18 years old provided written informed consent for participation. Any patient who had also been diagnosed with T1D prior to TPIAT was excluded from this analysis. Only patient surveys were included in this analysis (parent surveys completed on behalf of patients were excluded).

### 2.3. Diabetes-Related Quality of Life Measurements

The Pediatric Quality of Life Inventory 3.2 Diabetes Module™ (PedsQL™ DM) is a validated, standardized tool for evaluating DR-QOL in children and young adults with type 1 diabetes mellitus (T1D) [7]. This module utilizes a self-completed survey for children 5–18 years old, which contains 33 questions and can be used to generate a total score, a diabetes symptoms summary score (15 questions), and a diabetes management summary score (18 questions). The 18-question diabetes management summary score can be further divided into the following subscales: treatment barriers score (5 questions), treatment adherence score (6 questions), worry score (3 questions), and communication score (4 questions) [7].

Prior literature has suggested that differences in Pediatric Quality of Life Inventory 3.2 Diabetes Module™ (PedsQL™ DM) survey scores over time may not always represent clinically significant changes [8]. Utilizing the survey data to calculate the minimal clinically important difference (MCID) can help to identify whether survey results are clinically meaningful, such that a patient may perceive an improvement or worsening in DR-QOL [7]. Therefore, we also calculated MCID for the cohorts in this study and included this in our analysis.

### 2.4. TPIAT Group Quality of Life Survey Administration

The PedsQL™ DM survey was administered to assess DR-QOL after TPIAT. PedsQL™ DM scores were evaluated in a total of 46 children (<20 years old) who underwent TPIAT and completed at least one survey at the follow-up intervals. PedsQL™ DM data were collected at 3, 6, 9, 12, 18, and 24 months post-TPIAT. If a patient was <5 years old at the time of the survey, the parent completed the survey for the patient, so these scores were excluded. Of note, fewer participants were available for inclusion at 24 months post-TPIAT, as they had not yet reached this interval at the time of data collection.

### 2.5. Selection of the T1D Control Group and Quality of Life Survey Administration

Selection parameters based upon patient age of diabetes onset and sex were used to filter out a cohort of patients with T1D that was demographically similar to the TPIAT group. There were 164 patients who met the filter criteria and had at least one survey within the period of interest (±1 month of a time interval for TPIAT survey data). Of these, there were 52 and 58 patients who completed a survey at the 12-month and 24-month postdiagnosis time intervals, respectively. The use of this prior clinical data for this retrospective analysis was approved by the Cincinnati Children's Institutional Review Board.

The PedsQL™ DM Management Summary questions had been previously administered to patients with T1D at routine clinic appointments, typically at the beginning of the visit. The purpose of the survey during T1D clinic appointments was to help providers identify diabetes management issues and facilitate conversations to optimize clinical care. The diabetes symptom component of the survey (15 questions) had not been utilized during these T1D visits to lessen survey fatigue and clinic visit duration.

### 2.6. Statistical Analysis

Data were analyzed using SAS®, version 9.4 (SAS Institute, Cary, NC). Depending on distributions, continuous data were summarized as mean ± standard deviation or median with interquartile range (IQR: 25^th^–75^th^ percentiles). Categorical data were summarized as frequency counts and percentages. To analyze PedsQL™ DM score changes over time, generalized linear mixed models with repeated measures were used. Since the T1D patients often did not take the survey at exact intervals, the closest PedsQL™ DM survey that was ± 1 month from the target time after T1D diagnosis was used. For continuous data, *t*-tests or Wilcoxon–Mann–Whitney tests were used for comparisons between groups. For categorical data, the chi-square or Fisher's exact tests, as appropriate, were used for group comparisons. If more than half of the values were missing for a subscale or total score, the mean scores were not calculated for that subscale or total score, per the survey instructions [7]. A *P* < 0.05 was considered statistically significant. MCID scores were calculated from our data using the standard deviation multiplied by the square root of 1-Cronbach's*α*, as demonstrated by the following formula:

(1) $$\begin{array}{c} SEM=SD*\sqrt{\left(1-\alpha \right)}\text{.} \end{array}$$

## 3. Results

There were 46 patients in the TPIAT group and 164 patients in the T1D comparison group. The mean age at TPIAT was 12.6 ± 4.7 years and 67% were female. The mean age at T1D diagnosis was 11.2 ± 3.2 years and 69% were female (Table 1). In the TPIAT group, the median islet equivalent per kg (IEQ/kg) was 6,166 (IQR: 4204–7566). After 24 months, median hemoglobin A1c (HbA1c) was 6.6% (IQR: 5.7–7.9), and the rate of insulin independence was 42% (15/36) (Table 2).

**Figure 1:**
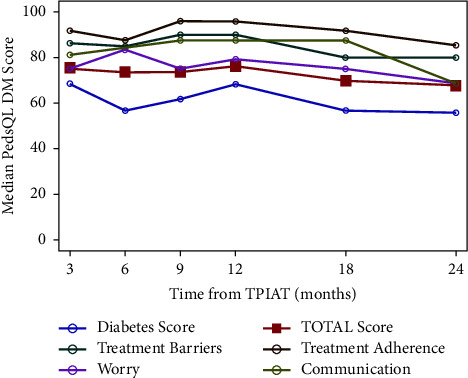
Median PedsQL DM scores the first two years after TPIAT.

**Figure 2:**
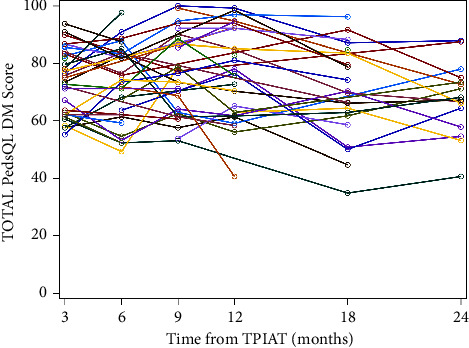
Total PedsQL DM scores of all participants the first two years after TPIAT.

Using 3-month post-TPIAT scores as a baseline, the diabetes symptoms summary score and the total score decreased (worsened) and reached MCID at 24 months post-TPIAT (diabetes symptoms summary: mean 65 to 57 *p* = 0.03; total score: mean 74 to 68 *p* < 0.05), (Tables3 and 4, Figures 1 and 2). The diabetes management subscales and management summary scores did not reach MCID at 24 months post-TPIAT. No mean scores reached MCID at 12 months, suggesting no clinically important differences were found at that interval.

When performing a subanalysis of the TPIAT group based upon insulin use at 24 months, ongoing insulin dependence (*n* = 11) resulted in a significantly lower (worse) diabetes symptom summary score (*p*=0.03) than was found in the insulin-independent cohort (*n* = 5) **(**Table 5**)**. The total score (*p*=0.09) and treatment barriers subscale (*p*=0.06) trended towards significance. Due to small sample sizes, MCID thresholds were not compared in the subanalysis.

Compared to the T1D cohort, the diabetes management summary score was not significantly different for the TPIAT patients at either 12 or 24 months (Table 6). Diabetes management subscales showed those 12 months after diabetes onset, only the treatment barriers subscale was statistically different between groups. In this subscale, the score was higher (better) in children 12 months post-TPIAT compared to children with T1D (*p* < 0.01). Importantly, eight of the 32 TPIAT patients were off insulin treatment at 12 months post-TPIAT. A subanalysis found that even the TPIAT patients still on insulin had higher (better) treatment barrier scores at 12 months than the T1D group (*p* < 0.05). By 24 months after TPIAT or T1D onset, the PedsQL DM management summary score and subscales were not significantly different between the TPIAT and T1D cohorts (all *p* > 0.2, Table 6).

The post-TPIAT and T1D MCIDs could be compared between groups for the management subscales. At the 12-month mark, MCIDs were generally similar between the two groups, with the exception that the TPIAT group had a higher MCID (11.04) on the communication subscale compared to the T1D group (7.60). At the 24-month mark, the communication MCID remained higher for TPIAT (11.73) than T1D (6.33) (Tables 4–7).

## 4. Discussion

This study expands on the previous literature evaluating diabetes-specific quality of life outcomes after TPIAT. Here, we report novel data on PedsQL™ DR-QOL measures in youth status post-TPIAT. This study is also the first to directly compare DR-QOL between children who have undergone TPIAT and children with new-onset T1D.

The PedsQL™ DM total score, mostly attributable to the diabetes symptoms score, decreased over time in youth who underwent TPIAT. Furthermore, the diabetes symptoms score and total scores after TPIAT reached the MCID threshold from 3 months to 24 months, suggesting a clinically meaningful difference in DR-QOL over this interval. This result was somewhat unexpected, as one might anticipate diabetes symptoms post-TPIAT to remain stable, or even improve, as patients become more experienced with diabetes management, and as some become insulin independent. It is not surprising that insulin-independence appears to be protective, as the diabetes symptoms score was significantly better in participants who were insulin-independent at 24 months (*p*=0.03), and the total score trended toward significantly higher scores (*p*=0.09) in the subanalysis of the TPIAT group (*n* = 5 off insulin and *n* = 11 on insulin). Thus, we presume that the worsening DR-QOL score over time is primarily attributable to the participants who remained insulin dependent. The subanalysis was underpowered for a full comparison, and a future investigation into DR-QOL in TPIAT, based upon insulin status, is needed.

It is possible that the worsening scores post-TPIAT, particularly in those who remained insulin dependent, may be related to patient expectations. Youth who undergo TPIAT may anticipate that the engrafted islet cells will eventually function adequately, which may be a protective factor for enhanced quality of life in the early months after the procedure. These youth may have the perspective that diabetes management is not as burdensome if they expect treatment duration for a limited time, compared to youth with T1D who expect that the same level of management will be lifelong. As time passes, particularly into the second year after TPIAT, hope for insulin independence may wane for some, thus creating additional perceived burden related to diabetes management. This highlights the necessity for clear and thorough preoperative counseling, and importantly, the need to include the patients in these discussions in a format that is age-appropriate.

The direct cross-sectional comparison of PedsQL™ DM Management scores between the post-TPIAT cohort and the T1D cohort showed that the scores were mostly similar between groups at 24 months. These similarities occurred despite a relatively large proportion (42%) of the post-TPIAT group who had become insulin independent, and the post-TPIAT group having a median HbA1c in goal range (<7%). Although we did not have data available in our T1D group to directly compare the diabetes symptoms scores, a systematic review evaluating QOL in children with T1D using either multiple daily injections or continuous subcutaneous insulin infusion regimen found neither group with T1D demonstrated a significant change in healthcare-related quality of life over time [9]. This contrasts with the diabetes symptoms and total scores in our TPIAT cohort, which decreased over the first 24 months.

Our data comparing TPIAT and T1D group scores did not support our initial hypothesis, that TPIAT DR-QOL scores would be higher (better) than the T1D cohort scores. It is possible that even though many in the post-TPIAT cohort were insulin independent, all of them were still expected to closely monitor blood glucose. Post-TPIAT, families and clinicians are tasked with trying to achieve extremely rigorous glucose control to protect the engrafted islet cells long-term. This extra vigilance may increase the burden of early diabetes management in this group. Additionally, all children post-TPIAT were started on insulin pump and continuous glucose monitor (CGM) therapy, whereas the standard approach for new onset T1D was to initially start on fingerstick glucose monitoring and a multiple daily injection regimen. In general, many children diagnosed with T1D will transition to utilize more advanced technology over the next 1-2 years. Perhaps this later transition contributed to a perception of improved quality of life in the T1D group, counterbalancing the perceived quality of life benefit from decreasing insulin requirements in the TPIAT group. Supporting this premise, the recent literature suggests technology utilization is associated with improved quality of life measures [10–12].

The calculated MCID management subscale scores for the TPIAT group ranged between 6 and 12, and except for the communication MCID, were similar to the T1D group. The management subscale MCIDs for the T1D group were generally consistent with past studies of T1D [8]. These numbers suggest that relatively large shifts in subscale scores (improvement or worsening), particularly in communication score for post-TPIAT patients, may be needed to detect changes that are apparent to children and their caregivers. The summary and total MCID scores could not be calculated in the T1D group but ranged from 3–6 in the TPIAT group. This indicates that smaller differences are needed to detect clinically meaningful changes for the summary and total scores.

Although this study is novel, it has some limitations. It is single-centered, which may make the data less generalizable. The data included in this analysis also only extend to the first two years after diagnosis, so later trends in quality of life after TPIAT cannot yet be determined. This dataset is limited by the lack of the diabetes symptoms score data in the T1D group, preventing a direct comparison of the symptoms summary score, as well as the total score, between the TPIAT and T1D cohorts. Another limitation is that the PedsQL™ DM has not been validated specifically for use in post-TPIAT diabetes population. However, the mechanism of diabetes post-TPIAT is effectively the same as in T1D: insulin deficiency. In addition, the PedsQL™ DM has been used and validated to assess QOL in youth with type 2 diabetes [8, 13], indicating broader utility than only T1D for evaluating symptoms of diabetes in youth. Last, the aim of this study was to evaluate DR-QOL specifically. The PedsQL™ DM survey cannot be generalized to overall healthcare-related quality of life, which might include improvement in pain or functional impairment. The prior literature has demonstrated improvement in components of physical and emotional quality of life for patients undergoing TPIAT [5]. Thus, in addition to DR-QOL, other facets of quality of life must also be considered when counseling patients.

## 5. Conclusions

This study provides valuable data for healthcare providers, patients, and families as they consider surgical management for ARP or CP. The data suggest that DR-QOL worsens over the first 24 months after TPIAT, and DR-QOL management scores are generally similar between TPIAT and T1D two years after diabetes onset. Further studies evaluating DR-QOL in youth over longer intervals will be necessary to help us understand the longer-term impact of TPIAT on quality of life. Both short-term and long-term DR-QOL data provide essential context for helping us to optimally counsel and manage patients with CP or ARP. Importantly, the data also suggest that insulin independence is likely to be protective for higher DR-QOL after TPIAT. This highlights the importance of advancing research and clinical improvement efforts, aimed at helping these individuals achieve insulin independence and experience a better quality of life.

## Figures and Tables

**Table 1:** Patient characteristics.

	TPIAT patients	T1D comparison group
	*N* = 46	*N* = 164
Age at TPIAT or T1D diagnosis (years)	12.6 ± 4.7 3.8–19.8)	11.2 ± 3.2 (3.9–19.7)
Sex (female)	31 (67%)	113 (69%)
Race		
White	44 (96%)	139 (85%)
Black/African American	0 (0%)	23 (14%)
Other	2 (4%)	2 (1%)
Ethnicity (non-Hispanic)	45 (98%)	161 (98%)

Data presented as mean ± SD (min–max) or *n* (%).

**Table 2:** Additional TPIAT patient data.

	TPIAT patients
	*N* = 46
BMI percentile	73.4 (45.4–93.0)
IEQ/kg	6166 (4204–7566)
Diagnosis	
ARP	3 (7%)
CP	43 (93%)
TDD/kg-12 months post-TPIAT	0.2 (0.0–0.4) *n* *=* 43
Off	12/43 (28%)
<0.3 TDD/kg	13/43 (30%)
≥0.3 TDD/kg	18/43 (42%)
HbA1c (%)-12 months post-TPIAT	6.4 (5.6–7.8) *n* *=* 43
TDD/kg- 24 months post-TPIAT	0.2 (0.0–0.5) *n* *=* 36
Off	15/36 (42%)
<0.3 TDD/kg	4/36 (11%)
≥0.3 TDD/kg	17/36 (47%)
HbA1c (%)-24 months post-TPIAT	6.6 (5.7–7.9) *n* *=* 33

Data presented as mean ± SD (min–max) or median (25^th^–75^th^ percentile) or *n* (%). ARP: acute recurrent pancreatitis; BMI: body mass index; CP: chronic pancreatitis; IEQ/kg: islet equivalents per kilogram; TDD/kg: total daily dose of insulin per kilogram; HbA1c: hemoglobin A1c.

**Table 3:** TPIAT PedsQL DM scores over time after TPIAT.

	3 month	6 month	9 month	12 month	18 month	24 month	*P*value (over 24 months)
	*N* = 36	*N* = 29	*N* = 29	*N* = 29	*N* = 21	*N* = 16	
Diabetes symptoms summary score	64.6 ± 14.3	61.9 ± 15.7	66.3 ± 17.1	67.5 ± 20.6	59.3 ± 18.7	56.5 ± 12.9 *¥*	**0.03**
Treatment barriers score	85.6 ± 13.6	84.7 ± 13.6	84.0 ± 16.0	86.4 ± 14.0	77.3 ± 20.2	77.5 ± 18.3	0.07
Treatment adherence score	85.8 ± 14.2	87.2 ± 11.1 *n* *=* *27*	89.9 ± 12.4	88.8 ± 13.0	86.7 ± 13.8	85.8 ± 11.6	0.90
Worry score	74.0 ± 23.7	73.0 ± 27.6 *n* *=* *27*	73.3 ± 27.9	75.0 ± 25.5 *n* *=* *28*	68.3 ± 30.1 *n* *=* *20*	67.2 ± 24.9	0.19
Communication score	76.9 ± 21.8	77.0 ± 20.8 *n* *=* *28*	81.7 ± 22.3	82.1 ± 24.1	75.6 ± 28.0	71.5 ± 25.6	0.70
Diabetes management summary score	80.6 ± 11.9	80.7 ± 15.0 *n* *=* *28*	82.2 ± 15.8	83.0 ± 13.8	77.2 ± 19.4	75.5 ± 15.3	0.24
Total score	74.0 ± 11.0	72.4 ± 13.5	75.8 ± 14.0	76.6 ± 15.7	69.7 ± 16.3	67.8 ± 12.3 *¥*	**0.049**

Data presented as means ± standard deviations. ^*∗*^only ≥5 years old filled out by patient. *¥* Average score difference exceeded minimal clinically important differences (MCID) over interval. Bold values meet criteria for statistical significance (*p* value <0.05).

**Table 4:** TPIAT patient MCID scores.

		Mean ± SD	*α*	MCID
12 months	Diabetes symptoms summary	67.5 ± 20.6 *n* *=* 29	0.87	7.43
	Barriers	86.4 ± 14.0 *n* *=* *29*	0.66	8.16
	Adherence	88.8 ± 13.0 *n* *=* *29*	0.64	7.80
	Worry	75.0 ± 25.5 *n* *=* *28*	0.82	10.82
	Communication	82.1 ± 24.1 *n* *=* *29*	0.79	11.04
	Diabetes management summary	83.0 ± 13.8 *n* *=* *29*	0.86	5.16
	Total score	76.6 ± 15.7 *n* *=* *29*	0.92	4.44

24 months	Diabetes symptoms summary	56.5 ± 12.9 *n* *=* *16*	0.87	4.65
	Barriers	77.5 ± 18.3 *n* *=* *16*	0.66	10.67
	Adherence	85.8 ± 11.6 *n* *=* *16*	0.64	6.96
	Worry	67.2 ± 24.9 *n* *=* *16*	0.82	10.56
	Communication	71.5 ± 25.6 *n* *=* *16*	0.79	11.73
	Diabetes management summary	75.5 ± 15.3 *n* *=* *16*	0.86	5.72
	Total score	67.8 ± 12.3 *n* *=* *16*	0.92	3.48

Management summary = barriers, adherence, worry, and communication subscores.

**Table 5:** PedsQL DM scores TPIAT group subanalysis: on or off insulin.

	TPIAT *N* = 32		*P* value
	On insulin at 12 months	Off insulin at 12 months	
	*N* = 21	*N* = 8	
12 months			
Diabetes symptoms summary	66.1 (53.3–73.3) *n* *=* 21	81.7 (44.2–94.2) *n* *=* *8*	0.48
Treatment barriers	90.0 (75.0–100) *n* *=* 21	100.0 (80.0–100) *n* *=* 8	0.14
Treatment adherence	91.7 (75.0–100) *n* *=* 21	95.8 (79.2–100) *n* *=* 8	0.96
Worry	70.8 (45.8–100) *n* *=* 20	91.7 (70.8–100) *n* *=* 8	0.25
Communication	87.5 (75.0–100) *n* *=* 21	96.9 (84.4–100) *n* *=* 8	0.37
Diabetes management summary	79.7 (71.3–94.4) *n* *=* 21	89.4 (81.6–99.5) *n* *=* 8	0.23
Total score	75.8 (62.9–84.8) *n* *=* 21	88.7 (64.8–95.8) *n* *=* 8	0.35

	On insulin at 24 months	Off insulin at 24 months	
	*N* = 11	*N* = 5	

24 months			
Diabetes symptoms summary	53.3 (48.3–58.3) *n* *=* 11	71.4 (63.3–73.3) *n* *=* 5	0.03
Treatment barriers	75.0 (55.0–85.0) *n* *=* 11	100.0 (80.0–100) *n* *=* 5	0.12
Treatment adherence	83.3 (75.0–91.7) *n* *=* 11	91.7 (90.0–100) *n* *=* 5	0.06
Worry	62.5 (50.0–75.0) *n* *=* 11	75.0 (50.0–100) *n* *=* 5	0.52
Communication	68.8 (37.5–100) *n* *=* 11	81.3 (50.0–100) *n* *=* 5	0.73
Diabetes management summary	75.5 (60.1–84.8) *n* *=* 11	74.7 (72.9–100) *n* *=* 5	0.31
Total score	66.7 (54.5–72.7) *n* *=* 11	75.0 (67.2–87.5) *n* *=* 5	0.09

Data presented as median (25^th^–75^th^ percentiles).

**Table 6:** PedsQL DM management scores TPIAT vs T1D.

	TPIAT	T1D	*P* value
	*N* = 32	*N* = 98	
12 months			
Treatment barriers	90.0 (80.0–100) *n* *=* 29	80.0 (60.0–90.0) *n* *=* 52	0.004
Treatment adherence	95.8 (75.0–100) *n* *=* 29	87.5 (81.2–95.8) *n* *=* 52	0.14
Worry	79.2 (50.0–100) *n* *=* 28	66.7 (54.2–83.3) *n* *=* 52	0.13
Communication	87.5 (75.0–100) *n* *=* 29	81.3 (62.5–96.9) *n* *=* 52	0.20
Management summary	82.4 (73.3–95.8) *n* *=* 29	79.0 (66.7–88.1) *n* *=* 52	0.06

24 months			
Treatment barriers	80.0 (60.0–95.0) *n* *=* 16	77.5 (65.0–90.0) *n* *=* 58	0.96
Treatment adherence	85.4 (79.2–95.8) *n* *=* 16	91.7 (79.2–95.8) *n* *=* 58	0.98
Worry	68.8 (50.0–87.5) *n* *=* 16	75.0 (50.0–91.7) *n* *=* 58	0.57
Communication	68.8 (50.0–100) *n* *=* 16	81.3 (68.8–100) *n* *=* 58	0.28
Management summary	75.1 (64.2–87.6) *n* *=* 16	80.6 (69.6–92.7) *n* *=* 58	0.41

Data presented as median (25^th^–75^th^ percentiles). Higher score indicates better quality of life.

**Table 7:** T1D patient MCID scores.

		Mean ± SD	*α*	MCID
12 months	Barriers	75.5 ± 16.8 *n* *=* 52	0.64	10.08
	Adherence	87.0 ± 10.6 *n* *=* 52	0.61	6.62
	Worry	67.5 ± 21.1 *n* *=* 52	0.72	11.17
	Communication	77.3 ± 22.9 *n* *=* 52	0.89	7.60

24 months	Barriers	77.2 ± 16.8 *n* *=* 58	0.64	10.08
	Adherence	86.1 ± 13.2 *n* *=* 58	0.61	8.24
	Worry	71.5 ± 23.4 *n* *=* 58	0.72	12.38
	Communication	80.2 ± 19.1 *n* *=* 58	0.89	6.33

## Data Availability

The data used to support the findings of this study are available upon request. Some of the data contained in this manuscript were previously presented as an oral presentation at the 2020 American Diabetes Association Meeting with an abstract submitted under the same title https://diabetesjournals.org/diabetes/article/69/Supplement_1/120-OR/60898/120-OR-Diabetes-Related-Quality-of-Life-Assessment.
